# Key factors associated with malaria infection among patients seeking care through the public sector in endemic townships of Ayeyarwady Region, Myanmar

**DOI:** 10.1186/s12936-022-04088-8

**Published:** 2022-03-15

**Authors:** Jillian Dunning, Nang Khaing Zar Aung, Abigail Ward, Moe Moe Aye, Christopher Lourenço, Sarah Gallalee, Stephen Lavenberg, Arnaud Le Menach, Myat Min Tun, Aung Thi

**Affiliations:** 1 Clinton Health Access Initiative, Yangon, Myanmar; 2 Clinton Health Access Initiative, Boston, MA USA; 3 Clinton Health Access Initiative, Vientiane, Laos; 4 Myanmar Vector Borne Disease Control Program, Ministry of Health and Sports, Nay Pyi Taw, Myanmar

**Keywords:** Myanmar, Malaria, Ayeyarwady, LLIN, Rapid diagnostic test, Forest transmission, Community health worker, Malaria elimination

## Abstract

**Background:**

Ayeyarwady Region in Myanmar has made significant progress towards malaria elimination, with cases decreasing from 12,312 in 2015 to 122 in 2019. As transmission declines, malaria becomes increasingly focalized both in geographic hotspots and among population groups sharing certain risk factors. Developing a thorough profile of high-risk activities associated with malaria infections is critical to ensure intervention approaches are evidence-based.

**Methods:**

A test-negative study was conducted from September 2017 to May 2018 in Ngaputaw, Pathein and Thabaung townships in Ayeyarwady Region. Patients that presented to selected public facilities or community health volunteers with fever answered survey questions on demographic and behavioural risk factors, including exposure to malaria interventions, and were assigned to case and control groups based on the result of a malaria rapid diagnostic test. A random-effects logistic regression model adjusted for clustering at the facility level, as well as any variables along the causal pathway described by a directed acyclic graph, was used to determine odds ratios and association with malaria infections.

**Results:**

A total of 119 cases and 1744 controls were recruited from 41 public facilities, with a mean age of 31.3 and 63.7% male. Higher risk groups were identified as males (aOR 1.8, 95% CI 1.2–2.9) and those with a worksite located within the forest (aOR 2.8, 95% CI 1.4–5.3), specifically working in the logging (aOR 2.7, 95% CI 1.5–4.6) and rubber plantation (aOR 3.0, 95% CI 1.4–6.8) industries. Additionally, links between forest travel and malaria were observed, with risk factors identified to be sleeping in the forest within the past month (aOR 2.6, 95% CI 1.1–6.3), and extended forest travel with durations from 3 to 14 days (aOR 8.6, 95% CI 3.5–21.4) or longer periods (aOR 8.4, 95% CI 3.2–21.6).

**Conclusion:**

Malaria transmission is highly focalized in Ayeyarwady, and results illustrate the need to target interventions to the most at-risk populations of working males and forest goers. It will become increasingly necessary to ensure full intervention coverage of at-risk populations active in forested areas as Myanmar moves closer to malaria elimination goals.

**Supplementary Information:**

The online version contains supplementary material available at 10.1186/s12936-022-04088-8.

## Background

**Fig. 1:**
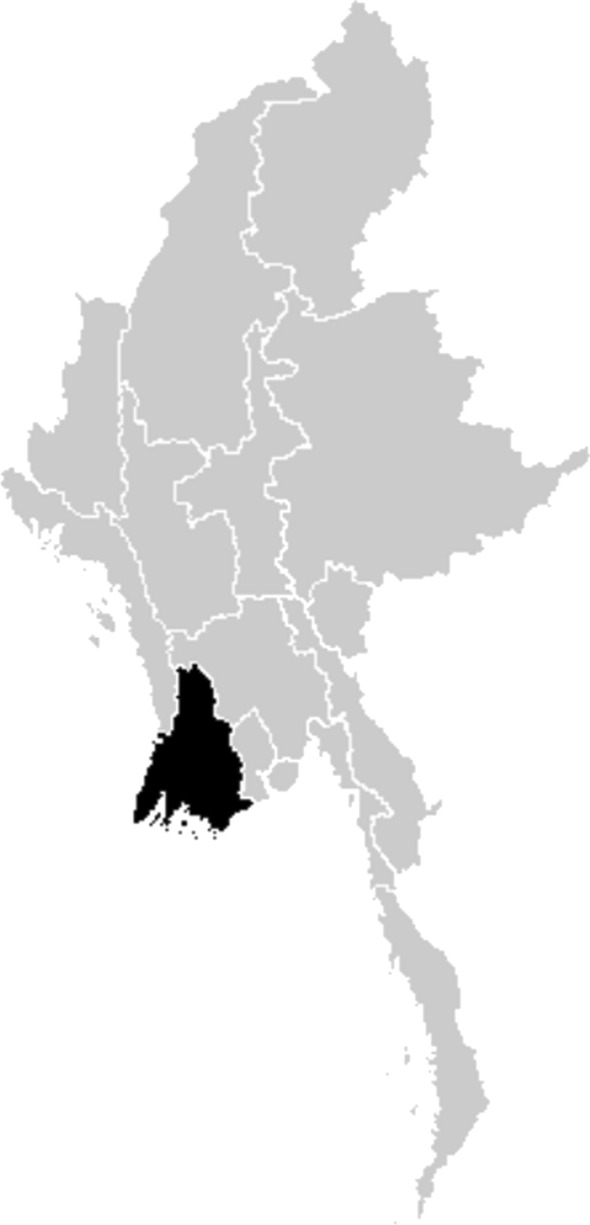
Ayeyarwady Region, Myanmar

Myanmar has achieved a substantial reduction in its national malaria burden, with a 69% decline between 2015 (182,616 malaria cases [1]) and 2019 (56,411 malaria cases [2]) and achieved notable progress towards malaria elimination goals. Ayeyarwady, a delta region in the southwest of Myanmar (Fig. 1), is indicative of this exponential decline at a regional scale. In 2015 the region accounted for 6.74% of national malaria burden with 12,312 confirmed cases [1], and in 2019 Ayeyarwady had decreased to a total of 122 (0.2% of annual cases in Myanmar) [3]. Within this timeframe, investments in increased deployment of integrated community malaria volunteers (ICMVs) in high-risk areas, coverage of vector control interventions, and the implementation of surveillance and case management guidelines have actively contributed to this significant case decline [1].

Cases are becoming increasingly focalized in certain geographies and population groups. In 2016, 96% of townships confirmed malaria cases in Ayeyarwady; in 2018, 73% of townships had reported at least one case [4]. Though malaria cases have decreased across the region, the three highest burden townships of Ngaputaw, Pathein and Thabaung reported malaria incidence in 2018 at a rate of 0.4 cases per 1000 population, four times the incidence rate of the regional total [5]. These contiguous townships share similar topography in levels of vegetation and forest cover, and reported a positivity rate of 18% in 2016, with 61% of cases identified at public health facilities and hospitals and 31% identified by integrated community malaria volunteers (ICMVs), with the remaining 8% diagnosed at private facilities [1].

To catalyze malaria elimination at the regional level, outreach to remaining hotspots is necessary to interrupt onward transmission. Refined at-risk population targets can ensure prioritization of intervention coverage and developing a thorough profile of high-risk activities associated with malaria infection in these areas is critical to ensure intervention approaches are evidence-based and cost-effective. Available surveillance data do not allow for the identification of specific demographic and behavioral risk factors, and prior attempts to characterize these groups through patient exit interviews and mobile migrant population questionnaires were limited in scale [6]. Additional evidence is required to understand the risk factors associated with forest-related transmission.

This study reports on identified risk factors among patients seeking care from public health facilities and ICMVs in the three highest burden townships of Ayeyarwady region. Identification of risk factors and effectiveness of interventions are provided, and characteristics that define population groups as higher risk may be used to target vector control and case management interventions.

## Methods

### Study population

Study participants included any patient over two years of age, presenting to one of 17 selected health facilities or 24 integrated community malaria volunteers (ICMVs) from September 1, 2017 through May 31, 2018. Patients presenting with a fever over 38 °C received a malaria test in accordance with national diagnosis guidelines, and study participants provided informed consent. Cases were defined as participants diagnosed with malaria confirmed by an SD BIOLINE Malaria Ag P.f/P.v rapid diagnostic test (RDT), and controls were participants with a negative RDT result.

### Study design

A test-negative study design was chosen to estimate malaria risk factors while maintaining key similarities in the control group including participation rates, diagnostic procedures, and information quality and completeness [7]. Test-negative studies recruit cases who attend a healthcare facility and test positive for a particular disease; controls are patients undergoing the same tests for the same reasons at the same healthcare facility and who test negative and has been posited as a separate type of study design, different from case–control studies as controls are not sampled from a wider source population. The design has the advantage of similar participation rates, information quality and completeness, referral/catchment areas, initial presentation, diagnostic suspicion tendencies, and preferences by doctors. Under certain assumptions, valid population odds ratios can be estimated with the test-negative design.

**Fig. 2:**
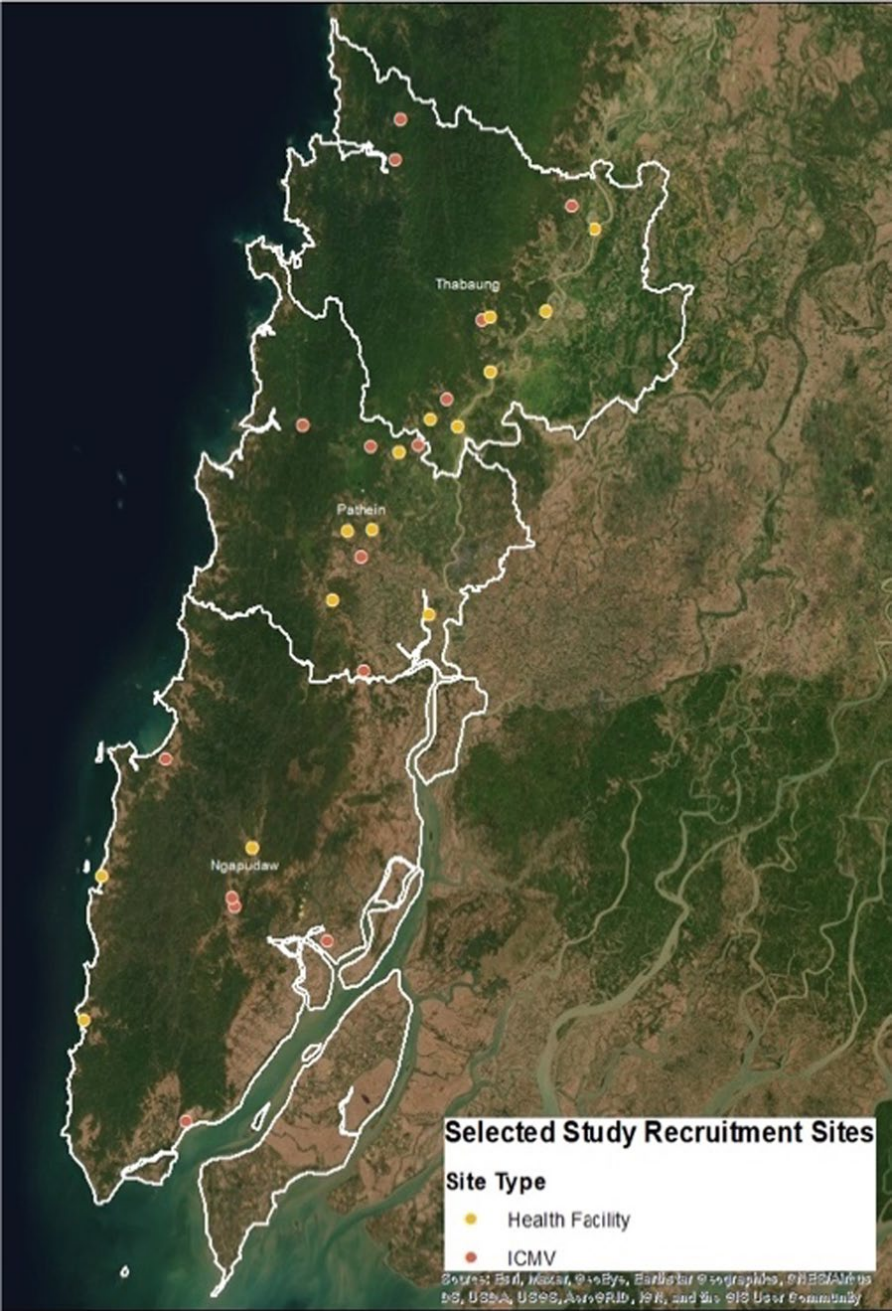
Selected Study Sites, HF and ICMV

The townships of Ngaputaw, Pathein and Thabaung were selected for the study as they represent some of the highest test positivity rates within Ayeyarwady Region and also share the highest forest cover within the larger Pathein district, where natural forest covered over 37% of its land area in 2010 [8]. With forest cover greatly influencing the transmission dynamics of malaria [9], the linkages between forest cover and malaria positivity rates in Ayeyarwady persist amid increasingly focalized malaria cases. As reported malaria cases sharply declined from 2016 to 2017, study sites were selected to prioritize recruitment of positive cases. The highest burden health facilities which had the operational capacity to administer the questionnaire in addition to providing routine services were selected (Fig. 2), determined from total reported cases from January 2016 to May 2017, which ranged from 9 to 793 [10]. Additionally, the eight highest burden villages with ICMVs were selected from each township.

In order to detect an odds ratio of 2, assuming an alpha risk of 5%, and a design effect of 1.5 to account for clustering at recruitment sites, a total of 625 study participants would be needed to achieve 80% power, with a case to control ratio of 1:4 [11]. To account for patient refusal, the targeted sample size was estimated at 132 cases and 525 controls. Study recruitment was timed to align with the highest transmission season in order to capture the most cases possible.

### Data collection

The survey was administered to cases and controls by basic health staff (BHS) at health facilities or by ICMVs at the point of care. Consent procedures were administered to all study participants to provide information on the study purpose and procedures, and participants were asked to provide written consent by signature. For participants younger than 18 years of age, written consent was provided by a parent or legal guardian. Written consent forms and surveys were administered by study coordinators in Burmese language. All BHS and ICMVs at selected study sites were trained on the study protocol, research ethics and informed consent, and the survey questionnaire prior to the enrollment of participants.

Individual data were collected including demographics (age, gender, occupation, education level, village of residence), whether village of residence is located in a forested area, occupation and worksite location (within or outside village), forest going behaviour (frequency and duration of travel, sleeping in the forest within the past month), habitual sleeping locations, preventive methods used including bed net type, and personal history and/or contacts with a history of malaria infection in the past year. Information captured on individual history of malaria infection as well as contacts within social and professional networks was reported by study participants, and this information was not cross-referenced against patient records at health facilities.

### Data processing

Responses were recorded on paper survey forms by BHS and ICMVs and completed forms were collected on a routine basis by study coordinators. Data were entered from paper forms into Microsoft Excel and study coordinators verified concordance between electronic and paper data. Data management, cleaning, and analysis were conducted in STATA 14.2 (StataCorp, College Station, TX).

### Data analysis

The odds of malaria infection associated with potential risk factors was assessed by logistic regression, including uncertainty expressed as 95% confidence intervals for each variable. A directed acyclic graph (DAG) was developed to assess the suitability of covariates for inclusion in the final multivariable models in order to minimize the magnitude of bias for covariate estimates on the risk of malaria infection and is included in Additional file 1 [12]. Each potential risk factor for malaria was assessed in a model including adjustment for potential confounders within the causal pathway. A binomial random-effects logit model was used for the adjusted models, with all chosen variables included as fixed effects and recruitment facility included as a random effect. A sub-group analysis of responses among participants who cited any forest travel was conducted with a binomial random-effects logit model on variables of interest related to forest travel and behaviors, as well as key occupation categories.

As many participants cited occupations across multiple industries, questionnaire responses were categorized into key occupations of interest in the adjusted models. Responses that included any work in the logging, rubber plantation, and farming industries were compared against all other reported occupations grouped together, as these industries represent outdoor work with remote locations and can include transitory workforces [13]. Missingness was assessed by question and the variable was removed from the adjusted model if missing data exceeded 10% of responses in case or control groups. For variables included in an adjusted model, missing data was included as a factor level in the model as its own response category.

## Results

Between September 2017 and May 2018, 122 RDT positive malaria cases were enrolled in the study, along with 1,807 RDT negative controls. Thirty participants were excluded from the analysis because RDT outcome information was missing in the completed survey form and could not be assigned to case or control groups. Thirty participants including three identified as cases were excluded from analysis due to missing informed consent forms, and 36 participants were excluded as they did not meet the age requirement (at least 2 years old). The final sample size was 119 cases and 1,744 controls (see Additional file 2 for flow diagram). The majority of study participants (60.3%) were recruited at the village level by ICMVs. Among RDT positive participants, 63.0% were diagnosed with *Plasmodium falciparum* infection, 33.6% with *Plasmodium vivax*, and 3.4% with mixed infection. Mean age among cases was 26.7 years (95% CI 24.0–29.4), slightly younger than control participants with a mean of 31.6 years (95% CI 30.9–32.4), though there were no significant differences in age groups between cases and controls. Occupations that included more than one industry were reported in 3.6% of participants, with 1.5% citing work in two or more of the logging, rubber plantation, and farming industries (Table 1). Of the 400 participants who reported any logging work as an occupation, 79.0% had slept in the forest within the past month.

**Table 1:** Demographic and behavioral characteristics of 1863 study participants, bivariate analysis odds ratio (OR) and 95% confidence intervals (95% CIs) for malaria infection against each covariate

	Cases	Controls	Unadjusted OR
	(n = 119)	(n = 1744)	(95% CI)
	N (%)	N (%)	
Age category			
Age 15–59*	100 (84.0)	1386 (79.5)	1
Age 2–14	17 (14.3)	271 (15.5)	0.9 (0.5–1.5)
Age 60–90	2 (1.7)	87 (4.9)	0.3 (0.1–1.3)
Sex			
Female*	27 (22.7)	579 (33.2)	1
Male	88 (73.9)	1,100 (63.1)	1.7 (1.1–2.7)
Missing	4 (3.4)	65 (3.7)	1.3 (0.4–3.9)
Township			
Ngaputaw*	54 (45.4)	480 (27.5)	1
Pathein	44 (36.9)	761 (43.6)	0.5 (0.3–0.8)
Thabaung	21 (17.6)	503 (28.8)	0.4 (0.2–0.6)
Education			
Illiterate/never attended school*	5 (4.2)	84 (4.8)	1
Able to read and write	11 (9.2)	352 (20.2)	0.5 (0.2–1.5)
Primary school	62 (52.1)	781 (44.8)	1.3 (0.5–3.4)
Middle school	37 (31.1)	376 (21.5)	1.6 (0.6–4.3)
High school	3 (2.5)	118 (6.8)	0.4 (0.1–1.8)
University student/graduate	1 (0.8)	20 (1.1)	0.8 (0.1–7.6)
Missing	0	13 (0.7)	–
Occupation			
Other occupation*	39 (32.8)	858 (49.2)	1
Any logging work	40 (33.6)	333 (19.1)	2.6 (1.7–4.2)
Any farming work	24 (20.2)	420 (24.1)	1.2 (0.7–2.1)
Any rubber plantation work	13 (10.9)	83 (4.7)	3.4 (1.8–6.8)
Two or more of the above	3 (2.5)	25 (1.4)	2.6 (0.8–9.1)
Missing	0	25 (1.4)	–
Recruitment point type			
Health facility*	55 (46.2)	684 (39.2)	1
ICMV	64 (53.8)	1060 (60.8)	0.7 (0.5 – 1.1)
Village located in the forest			
No*	56 (47.0)	855 (49.0)	1
Yes	58 (48.7)	816 (46.8)	1.1 (0.7–1.6)
Missing/don’t know	5 (4.2)	73 (4.2)	1.0 (0.4–2.7)
Worksite located in the forest (outside of home village)			
No*	27 (22.7)	811 (46.7)	1
Yes	91 (76.5)	894 (51.4)	3.0 (1.9–4.7)
Missing	1 (0.8)	33 (1.9)	0.9 (0.1–6.9)
Duration of forest travel			
No forest travel*	12 (11.5)	627 (41.0)	1
Less than 3 days	17 (16.3)	370 (24.2)	2.4 (1.1–5.1)
3–14 days	37 (35.5)	231 (15.1)	8.3 (4.2–16.3)
Longer than 14 days	36 (34.6)	210 (13.7)	8.9 (4.5–17.5)
Missing	2 (1.9)	91 (5.9)	1.1 (0.2–5.2)
Slept in the forest within the past month			
No*	25 (21.0)	953 (54.6)	1
Yes	90 (75.6)	681 (39.0)	5.0 (3.2–7.9)
Missing/don't know	4 (3.4)	110 (6.3)	1.4 (0.5–4.0)
Prevention methods used at home			
No preventive methods at home*	2 (1.7)	27 (1.5)	1
Preventive methods do not include bed nets (spatial/topical repellents, etc.)	8 (6.7)	127 (7.3)	0.8 (0.2–4.2)
Preventive methods include bed nets	93 (78.1)	1434 (82.2)	0.9 (0.2–3.7)
Missing/Don’t know	16 (13.4)	156 (8.9)	1.3 (0.3–6.3)
Preventive methods used in the forest			
No preventive methods in forest*	25 (23.3)	197 (17.6)	1
Preventive methods do not include bed nets (spatial/topical repellents, etc.)	12 (20.2)	132 (11.8)	0.7 (0.3–1.5)
Preventive methods include bed nets	61 (53.8)	706 (63.2)	0.6 (0.4–1.1)
Missing/don’t know	9 (8.4)	82 (7.3)	0.8 (0.4–1.9)
Type of net used while sleeping in the forest			
Conventional net*	34 (31.7)	301 (26.9)	1
LLIN/ITN	28 (26.2)	398 (35.6)	0.6 (0.3–1.0)
Hammock Net	0	3 (0.2)	–
Use another form of prevention	35 (32.7)	295 (26.4)	1.0 (0.6–1.7)
Don’t know	4 (3.7)	11 (0.9)	3.2 (0.9–10.6)
Missing	6 (5.6)	109 (9.7)	0.5 (0.2–1.2)
Self-reported malaria infection in the past year			
No one respondent knows infected*	59 (49.6)	1170 (67.1)	1
Respondent infected (or respondent and someone else among contacts)	34 (28.5)	71 (4.1)	9.5 (5.8–15.4)
Friends, family, coworkers, other contact infected (Respondent not infected)	17 (14.3)	277 (15.9)	1.2 (0.7–2.1)
Missing/don’t know	9 (7.6)	226 (12.9)	0.8 (0.3–2.9)

*CI *confidence interval, *ICMV*  integrated community malaria volunteer, *LLIN*  Long-lasting insecticidal net, *ITN*  Insecticide-treated net

*Baseline category

Bolded categories indicate ORs significant at p < 0.05

In bivariate analysis, confirmed malaria cases were more likely to be male (OR 1.7, 95% CI 1.1–2.7), work in logging (OR 2.6, 95% CI 1.7–4.2) or rubber plantations (OR 3.4, 95% CI 1.8–6.8), have a worksite located within the forest (OR 3.0, 95% CI 1.9–4.7), stay within the forest for a period longer than 3 days, and report that they had malaria within the past year (OR 9.5, 95% CI 5.8–15.4). To examine the potential impact of relapse on participant responses of past malaria among cases confirmed with *P. vivax* infection, a chi-square test of independence was used to determine any association between malaria infection species and self-reported malaria within the past year and did not find a statistically significant association. Refraining from forest travel showed a protective effect (OR 0.2, 95% CI 0.1–0.5). Table 1 shows the demographics of study participants included in the sample and bivariate odds ratios.

The final seven adjusted multivariable models identified significant risk groups as males (aOR 1.8, 95% CI 1.2–2.9) and those reporting a worksite located in the forest (aOR 2.8, 95% CI 1.4–5.3), particularly any work in the logging (aOR 2.7, 95% CI 1.5–4.6) and rubber plantation (aOR 3.0, 95% CI 1.4–6.8) industries. Individuals traveling to the forest for 3 to 14 days (aOR 8.6, 95% CI 3.5–21.4) and periods of 14 days or longer (aOR 8.4, 95% CI 3.2–21.6) are also at a significantly higher risk (Table 2).

**Table 2:** Adjusted odds ratios odds ratio (aOR) and 95% confidence intervals (95% CIs) for malaria infection against each covariate and adjustment for potential confounders

Exposure	aOR, CI
Age category^a^	
Age, 15–59*	1
Age, 2–14	0.8 (0.4–1.4)
Age, 60–90	0.4 (0.1–1.7)
Sex^a^	
Female*	1
Male	1.8 (1.2–2.9)
Missing	1.47 (0.5–4.6)
Education^b^	
Illiterate/never attended school*	1
Able to read and write	0.3 (0.1–1.1)
Primary school	0.9 (0.3–2.5)
Middle school	1.1 (0.4–2.9)
High school	0.26 (0.1–1.2)
University Student/graduate	0.4 (0.1–4.9)
Missing	1*
Occupation^c^	
Other occupation*	1
Any logging work	2.7 (1.5–4.6)
Any farming work	1.1 (0.6–2.0)
Any rubber plantation work	3.0 (1.4–6.8)
Two or more of the above	3.3 (0.8–12.8)
Missing	1*
Worksite located in the forest (outside of home village)^d^	
No*	1
Yes	2.8 (1.4–5.3)
Missing	2.7 (0.3–26.4)
Duration of forest travel^e^	
No forest travel*	1
Less than 3 days	2.4 (0.9–5.7)
3–14 days	8.6 (3.5–21.4)
Longer than 14 days	8.4 (3.2–21.6)
Missing	1.5 (0.3–7.3)
Slept in the forest within the past month^f^	
No*	1
Yes	2.6 (1.1–6.3)
Missing/don’t know	1.8 (0.2–12.4)
Preventive methods used in the forest^g^	
Preventive methods do not include bed nets*	1
Preventive methods include bed nets	0.6 (0.3–1.3)
No preventive methods	1.6 (0.7–3.7)
Missing/don’t know	2.6 (0.9–7.5)
Type of net used while sleeping in the forest^g^	
Conventional net*	1
LLIN/ITN	0.6 (0.3–1.1)
Hammock net	1*
Use another form of prevention	1.4 (0.7–2.7)
Don’t know	1.4 (0.4–4.6)
Missing	2.1 (0.7, 6.7)

*Baseline categories, aOR = 1* indicates too few observations

*CI* confidence interval, *aOR*  adjusted odds ratio, *ICMV* integrated community malaria volunteer, *LLIN* Long-lasting insecticidal net, *ITN*  Insecticide-treated net

Bolded categories indicate ORs significant at p < 0.05

^a^Model 1 (n = 1863): adjusted for point of care (random effect)

^b^Model 2 (n = 1863): adjusted for age, sex, and point of care

^c^Model 3 (n = 1863): adjusted for age and point of care

^d^Model 4 (n = 1863): adjusted for age, occupation and point of care

^e^Model 5 (n = 1863): adjusted for age, worksite in the forest, and point of care

^f^Model 6 (n = 1224): adjusted for age, duration of forest travel, worksite in the forest, and point of care

^g^Model 7 (n = 1224): adjusted for age, sleeping in the forest, and point of care

### Forest-going populations

A total of 1,224 respondents including 107 cases and 1117 controls (65% of total sample) reported any forest travel, with varied frequencies. Forest travel was defined as traveling through or staying in the forest outside of the home, in order to estimate the effect of longer-term forest related economic activity as well as increased mosquito exposure from sleeping outdoors or in temporary structures. Within the subset of forest goers, no significant differences were found between case and control groups in the use of preventive methods in the forest nor in the type of bed net used, after adjusting for age group, worksites located in the forest, duration of forest travel/sleeping in the forest, and point of care (Table 2).

## Discussion

Among those seeking care for fever in the public sector of malaria-endemic townships in Ayeyarwady Region, there are higher odds of malaria infection among those that are male, working in forested areas and in outdoor occupations of rubber plantations and logging, often traveling to the forest for extended periods and sleeping away from the home. Those with a history of malaria or have social and professional connections to those with a history of malaria also appear to be at higher risk, though the causal linkage to malaria infection cannot be directly established. Among forest travellers, regular use of preventive methods while in the forest and use of an insecticide-treated net did not significantly reduce the risk of malaria when adjusting for the effect of age, point of care, and sleeping in the forest.

Villages of residence for cases and controls were located around the forest fringe, and the catchment areas of selected health facilities include several registered worksites in the logging and agricultural industries. When asked for village of residence, several study participants reported the location or company name of their employer, suggesting lengthy periods of time away from home to pursue economic opportunities and worksite related travel in forested areas. Although there was no increased risk of malaria infection for those who lived in villages within the forest, there was an increased risk for individuals who had forest worksites. This suggests the characteristics of lodging and occupational behaviours and practices for forest work provide opportunity for malaria transmission. Travel in forested areas [14] and for occupational purposes [15, 16] among males [17, 18] have shown to be associated with malaria risk in other studies conducted in low endemic and elimination settings. Sleeping outside of the household has also been identified as a risk factor in other contexts [19, 20] and these results confirm the linkage to these occupational and forest travel components within the townships in Ayeyarwady, while also providing additional details on preventive method use specifically among the population of forest goers. Study results consistent with existing literature reinforce the potential impact of workplace-focused mechanisms such as expanding diagnosis services directly at work sites and ensuring access to prevention and treatment services among groups traveling and working in forested areas for extended periods of time.

A mix of intervention approaches may be required to ensure the most effective locally-tailored strategies are applied [21]. Higher malaria risk among those citing any work in logging and rubber plantation industries suggests the malaria cases that persist in Ayeyarwady Region are perpetuated by communities connected socially and professionally, and the most effective outreach methods to target connected groups should consider approaches proven successful in this context. Case contact tracing and high-risk group screening may facilitate the identification and treatment of connected malaria cases [22].

Findings in Myanmar indicate that passive ICMV testing has higher positivity rates compared to mobile clinics [23] and that active case detection in response to positive cases does not necessarily identify additional cases in low endemic areas [24]. Expanding health services provided by ICMVs in hard-to-reach areas [25] or within the catchment of forest worksites, emphasis on improved case management practices, and strong case and foci investigation and response within these connected communities would impact the acceleration of progress in malaria decline. Interventions that have been implemented in limited settings or have provided less evidence of effectiveness within the context of Southeast Asia such as mass drug administration [26, 27], stand-by presumptive treatment and chemoprophylaxis [28], topical repellents [29], insecticide treated clothing [30], and insecticide treated hammock nets [31] would not be interchangeable with core interventions around surveillance and case management. Study results indicate that targeting forest workers and ensuring the acceptability of interventions among this group is a critical component to an effective malaria elimination strategy.

The multivariable analysis found no associations between bed net usage and malaria infection at home, or among forest going populations. A substantial proportion of case participants (31.7%) reported use of a bed net that is not treated with insecticide in the forest though endemic areas continue to be targeted by LLIN campaigns. This prompts further questions around the distribution of LLINs among high-risk groups, as well as available options for regular re-treatment of bed nets. Information on additional malaria preventive methods (including topical and spatial mosquito repellents) in forested areas were not collected in this survey. Information around sleeping arrangements and behaviour during outdoor biting hours, including the feasibility and acceptability of preventive methods within the context of forest work, would improve the identification of potential exposures within this population and allow better targeting of vector control interventions and more effective malaria prevention.

There were several limitations as a result of study design. Survey administration was incorporated into routine health care and consequently may underrepresent characteristics of populations who do not seek treatment at health care facilities, which may include seasonal workers and migrant populations. In order to mitigate this potential effect, eight ICMVs were selected in addition to six health facilities in each study township, under the assumption that hard to reach populations would be more likely to present to a health worker at the village level. Additionally, study participant selection was limited to those over two years of age, which led to the exclusion of 36 participants from the study and impacts identification of risk factors among young children and generalizability to younger age groups. Survey administrators were routine health care providers who are responsible for providing bed nets and health promotion activities to the study communities. Consequently, participants may have felt the need to overstate use of malaria preventive methods and bed net usage. Finally, self-reported frequency and duration of forest travel as well as use of preventive methods could be affected by recall bias. The target sample size was not met, though study recruitment was focused on high-burden facilities within high-burden townships, and data collection was conducted during peak malaria season, which limited the representativeness of the study population to other geographies in Myanmar.

## Conclusion

This study identified several risk factors for malaria transmission that present a continuous challenge to elimination efforts. In this setting, sleeping in the forest in the past month, and a prior history of malaria infection were found to be the strongest risk factors for malaria. Ensuring that receptive areas, such as worksites where employees are sleeping in forested areas outdoors and overnight, are prioritized for intervention coverage could have an impact in the prevention of local malaria transmission. As a result of these findings, designing a package of interventions targeted to those that travel and work in and around the forest could catalyze progress toward elimination in Ayeyarwady Region.

## Supplementary Information

**Additional file 1.** Causal directed acyclic graph (DAG). The figure represents the causal diagram for the data.

**Additional file 2.** Study participant flow diagram.

## Data Availability

The data that support the findings of this study are not publicly available due to data protection from the Vector Borne Disease Control Program in Myanmar. Aggregate and anonymized data can be available from the corresponding author upon reasonable request and with permission of the Myanmar Vector Borne Disease Control Program, Ministry of Health and Sports.

## Abbreviations

aOR: Adjusted odds ratio
BHS: Basic health staff
CHAI: Clinton Health Access Initiative
CI: Confidence interval
DAG: Directed acyclic graph
GMS: Greater Mekong Subregion
HF: Health facility
ICMV: Integrated community malaria volunteer
ITN: Insecticide-treated net
LLIN: Long-lasting insecticidal net
NMCP: National Malaria Control Programme
OR: Odds ratio
RDT: Rapid diagnostic test
