# Supplementary material for: Key factors associated with malaria infection among patients seeking care through the public sector in endemic townships of Ayeyarwady Region, Myanmar
Source: Malar J. 2022 Mar 15;21:86. doi: 10.1186/s12936-022-04088-8 (PMC8922824; doi:10.1186/s12936-022-04088-8)
Supplement: Supplementary file 1 — Additional file 1. Causal directed acyclic graph (DAG). The figure represents the causal diagram for the data. [file 12936_2022_4088_MOESM1_ESM.docx]

# Additional File 1

Causal directed acyclic graph (DAG). The figure represents the causal diagram for the data.
