# Supplementary figures and images for: Key factors associated with malaria infection among patients seeking care through the public sector in endemic townships of Ayeyarwady Region, Myanmar
Source: Malar J. 2022 Mar 15;21:86. doi: 10.1186/s12936-022-04088-8 (PMC8922824; doi:10.1186/s12936-022-04088-8)

# Additional File 2

Study participant flow diagram


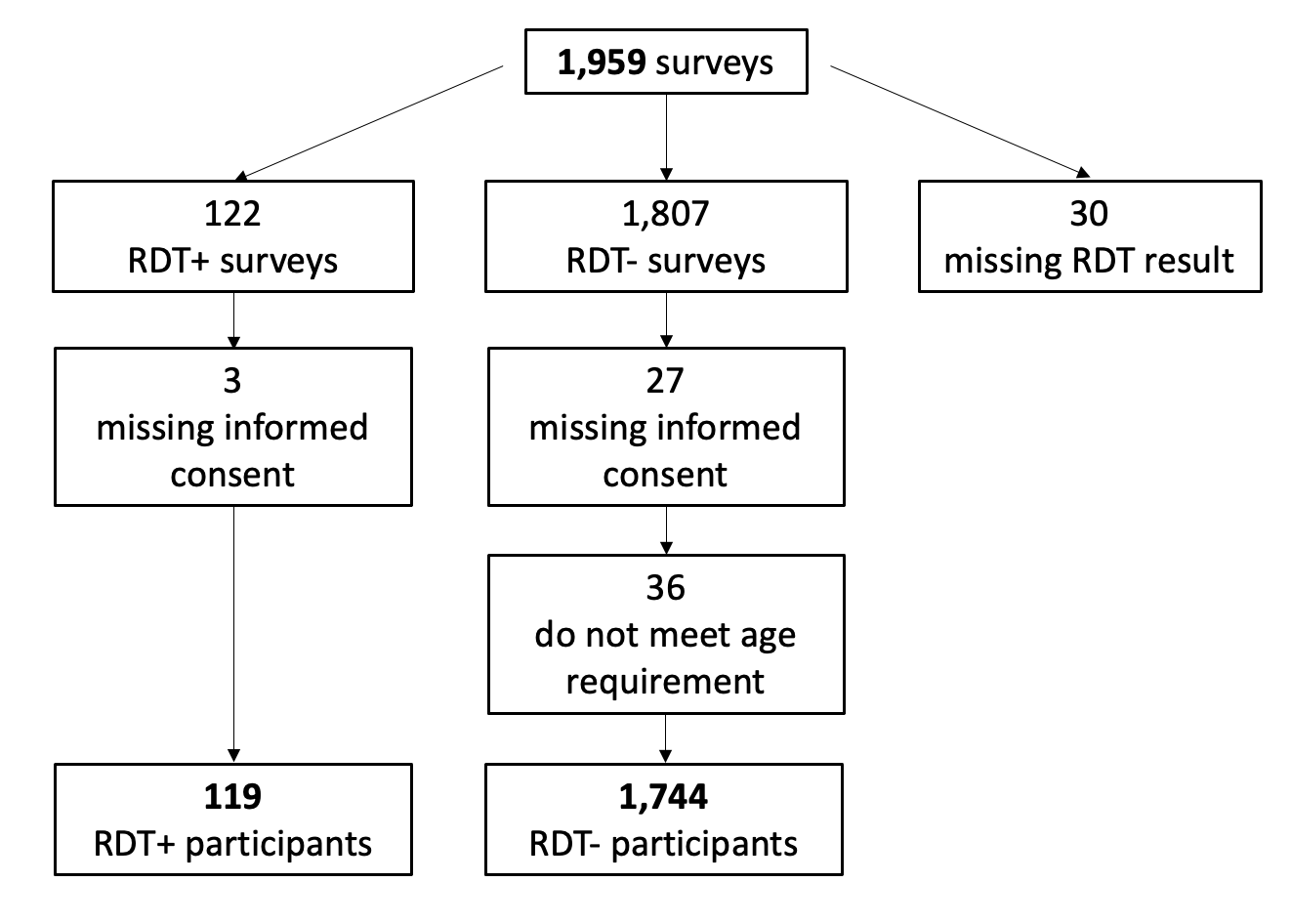

Supplement: Supplementary file 2 — Additional file 2. Study participant flow diagram. [file 12936_2022_4088_MOESM2_ESM.docx]
